# Associated abdominal injuries do not influence quality of care in pelvic fractures—a multicenter cohort study from the German Pelvic Registry

**DOI:** 10.1186/s13017-020-0290-x

**Published:** 2020-01-17

**Authors:** Markus A. Küper, Robert Bachmann, Götz F. Wenig, Patrick Ziegler, Alexander Trulson, Inga M. Trulson, Christian Minarski, Ruth Ladurner, Ulrich Stöckle, Andreas Höch, Steven C. Herath, Fabian M. Stuby, Working Group on Pelvic Fractures of the German Trauma Society

**Affiliations:** 1 BG Trauma Center, Department for Traumatology and Reconstructive Surgery, University of Tübingen, Schnarrenbergstraße 95, 72076 Tübingen, Germany; 2 Department of General, Visceral and Transplant Surgery, University Hospital Tübingen, Tübingen, Germany; 3 Department of Trauma Surgery, BG Trauma Centre Murnau, Murnau am Staffelsee, Germany; 4 Charité University Medicine Berlin, Center for Musculoskeletal Surgery, Berlin, Germany; 5 Department of Orthopedics, Trauma and Plastic Surgery, University of Leipzig, Leipzig, Germany; 6 Department of Trauma, Hand and Reconstructive Surgery, Saarland University Hospital, Homburg, Germany

**Keywords:** Pelvic trauma, Pelvic ring fracture, Acetabular fracture, Abdominal trauma, Postoperative reduction, Registry study

## Abstract

**Background:**

Pelvic fractures are rare but serious injuries. The influence of a concomitant abdominal trauma on the time point of surgery and the quality of care regarding quality of reduction or the clinical course in pelvic injuries has not been investigated yet.

**Methods:**

We retrospectively analyzed the prospective consecutive cohort from the multicenter German Pelvic Registry of the German Trauma Society in the years 2003–2017. Demographic, clinical, and operative parameters were recorded and compared for two groups (isolated pelvic fracture vs. combined abdominal/pelvic trauma).

**Results:**

16.359 patients with pelvic injuries were treated during this period. 21.6% had a concomitant abdominal trauma. The mean age was 61.4 ± 23.5 years. Comparing the two groups, patients with a combination of pelvic and abdominal trauma were significantly younger (47.3 ± 22.0 vs. 70.5 ± 20.4 years; *p* < 0.001). Both, complication (21.9% vs. 9.9%; *p* < 0.001) and mortality (8.0% vs. 1.9%; *p* < 0.001) rates, were significantly higher.

In the subgroup of acetabular fractures, the operation time was significantly longer in the group with the combined injury (198 ± 104 vs. 176 ± 81 min, *p* = 0.001). The grade of successful anatomic reduction of the acetabular fracture did not differ between the two groups.

**Conclusion:**

Patients with a pelvic injury have a concomitant abdominal trauma in about 20% of the cases. The clinical course is significantly prolonged in patients with a combined injury, with increased rates of morbidity and mortality. However, the quality of the reduction in the subgroup of acetabular fractures is not influenced by a concomitant abdominal injury.

**Trial registration:**

ClinicalTrials.gov, NCT03952026, Registered 16 May 2019, retrospectively registered

## Introduction

Pelvic fractures, as well as abdominal injuries, are severe injuries, which require a careful and interdisciplinary decision-making regarding the therapeutic regime. Main causes for both, pelvic fractures and abdominal injuries, are traffic accidents or falls from different heights. Especially the combination of pelvic fractures with abdominal injuries is often caused by a high-energy-trauma and may be life-threatening [1]. The mortality rates for both, pelvic fractures or abdominal injuries, are about 5–10% and are associated especially with hemodynamic instability [2–5].

Pelvic fractures can be subdivided into pelvic ring fractures and acetabular fractures. Due to the complex anatomy of the pelvic bones and the surrounding soft tissue, as well as the rarity of these fractures, the treatment of pelvic fractures can still be a challenge for the orthopedic surgeon. The optimal time-period for osteosynthetic stabilization of pelvic fractures, is determined by both, concomitant injuries and hemodynamic stability.

The aim of the osteosynthetic stabilization of pelvic ring injuries is the recreation of stability of the pelvic ring. The stability of the pelvic ring can be assessed by using the Tile-classification, where the integrity of the posterior pelvic ring is decisive for the stability of the entire pelvic ring [6]. Therefore, especially, Tile B or C injuries require surgical stabilization. Nowadays, this can be obtained by a percutaneous insertion of sacroiliac screws in most cases [7]. If additional stabilization of the anterior pelvic ring is necessary, there are other available approaches like open surgical procedures with plate osteosynthesis. However, open surgery often means a “second-hit,” so definitive treatment with a supraacetabular external fixator is also an option [8]

Regarding acetabular fractures, like in all articular fractures, the main goal of treatment is the anatomical reconstruction of the joint line to prevent the development of posttraumatic osteoarthritis. The classification of acetabular fractures follows the classification of Letournel and Judet. Factors that influence the decision of how to treat acetabular fractures besides age and co-morbidities are the fracture type, concomitant injuries, and the grade of dislocation especially in the main weight bearing zone of the hip joint, as well as the time interval between accident and surgical treatment [9]. Open reduction and plate osteosynthesis is the gold standard in the treatment of dislocated acetabular fractures to reconstruct the joint line. The quality of reduction is rated by the Matta score. Grade 1 (anatomical reconstruction) is defined as a residual fracture step-off < 2 mm, grade 2 (imperfect reduction) is defined as a residual fracture step-off of 2–3 mm, and grade 3 (poor reduction) is defined as a residual fracture step-off of > 3 mm [10]. A non-anatomical reduction of the acetabular surface leads to a shifting in the main pressure zone of the hip joint with consecutive biomechanical changes and the development of a posttraumatic osteoarthritis [11].

Whether a combined injury, consisting of pelvic fracture and abdominal injury, results in a worse postoperative outcome of the treated pelvic fracture, due to a possibly prolonged surgical treatment of the pelvic injury (after abdominal injuries have been treated), is not known yet and is under investigation [12, 13].

The present multicenter cohort study investigates the impact of an associated abdominal injury on the clinical course, the delay on the surgical treatment and the surgical outcome of acetabular fractures. Our main hypothesis was that an associated abdominal injury leads to a prolonged definitive surgical treatment of the pelvic fractures. The secondary hypothesis was that the prolonged surgical treatment leads to a worse reduction quality of acetabular fractures in patients with a combined abdominal and pelvic injury.

## Patients and methods

### Patient cohort

The GPR (German Pelvic Registry) is a prospective nationwide multicenter database with 30 participating hospitals. It was developed in 1991 by the Working Group “Pelvic Injuries” of the German Society for Traumatology (Deutsche Gesellschaft für Unfallchirurgie; DGU) in cooperation with the German Section of AO International in order to collect anonymized in-hospital data of patients with a pelvic ring and/or acetabular fracture [14]. The headquarter of the database is located at the Department of Trauma, Hand and Reconstructive Surgery of the Saarland University Hospital in Homburg/Saar and the Ethics Committee of the Chamber of Physicians of the Federal State of Saarland approved the GPR (No. 29/14). Data-management was done by MEMDoc, a specialist for clinical registries at the University of Bern in Switzerland. Eligibility criteria for enrollment into the registry are a pelvic ring and/or acetabular fracture and the informed consent of the patients. The follow-up is individually determined by the duration of in-hospital treatment due to the pelvic injury.

In this cohort study, the data from January 2003 to December 2017 were investigated retrospectively. The local Ethics Committee of the Eberhard-Karls-University in Tübingen, Germany, approved this cohort study (No. 968/2018BO2). A total of 16.359 patients with pelvic fractures were recorded correctly and completely with 3.335 (20.4%) suffering from an acetabular fracture.

The abdominal injury in the GPR was defined according to the criteria of the Injury Severity Scores (ISS) as an Abbreviated Injury Score (AIS abdomen) > 0.

### Evaluated parameters

The following parameters were transferred from the original Microsoft Excel database after transfer to SPSS Statistics 26.0® (IBM Corporation, Armonk, NY, USA) for further statistical analysis:

- Age
- Gender
- Injury Severity Score (ISS)
- Hemoglobin level (Hb) at admission
- Systolic blood pressure (RR) at admission
- Number of emergency stabilizations
- Number of definitive surgical stabilizations
- Time until emergency fracture stabilization (in minutes)
- Time until definitive stabilization (in days)
- Length of hospital stay (in days)
- Overall complication rate (except osteosynthesis-associated complications)
- Rate of osteosynthesis-associated complications
- Mortality

The following complications were recorded:

- Bleeding events
- Thromboembolic events
- Surgical site infection (superficial and deep)
- Fracture-associated neurologic complications (preoperatively existing)
- Iatrogenic neurologic complication
- Pulmonary complications
- Cardiac complications
- Multi organ failure

The following osteosynthesis-associated complications were recorded:

- Implant loosening
- Implant failure
- Secondary displacement of the fracture after fixation

To investigate the quality of surgery, in a subgroup of isolated acetabular fractures, the following procedural parameters with a focus on the postoperative reduction quality were evaluated:

- Duration of surgery (in minutes)
- Blood loss (in milliliter)
- Preoperative maximal fracture step-off (in millimeter)
- Postoperative maximal fracture step-off (in millimeter)
- Reduction quality according to Matta classification [10]

The acetabular fracture step-offs pre- and postoperatively were recorded by experienced acetabular trauma surgeons in the respective operating hospital during the inpatient treatment of the patients, and the maximum steps were entered in the prospective database. Both, pre- and postoperative maximal fracture step-off were recorded using either plain X-ray of the pelvis (including iliac/obturator oblique views) or CT-Scan if available.

### Statistics

The data are presented as mean ± standard deviation unless stated otherwise. Differences between the mean values of the groups were calculated using the two-sided paired Student’s *t* test. Differences between the frequencies were calculated using the Mann-Whitney *U* test. A *p* value < 0.05 was considered as statistically significant.

All statistics were calculated using SPSS Statistics® with help of Mrs. Inka Rösel (Institute for Clinical Epidemiology and Applied Biometry, University of Tübingen, Germany.

## Results

**Fig. 1:**
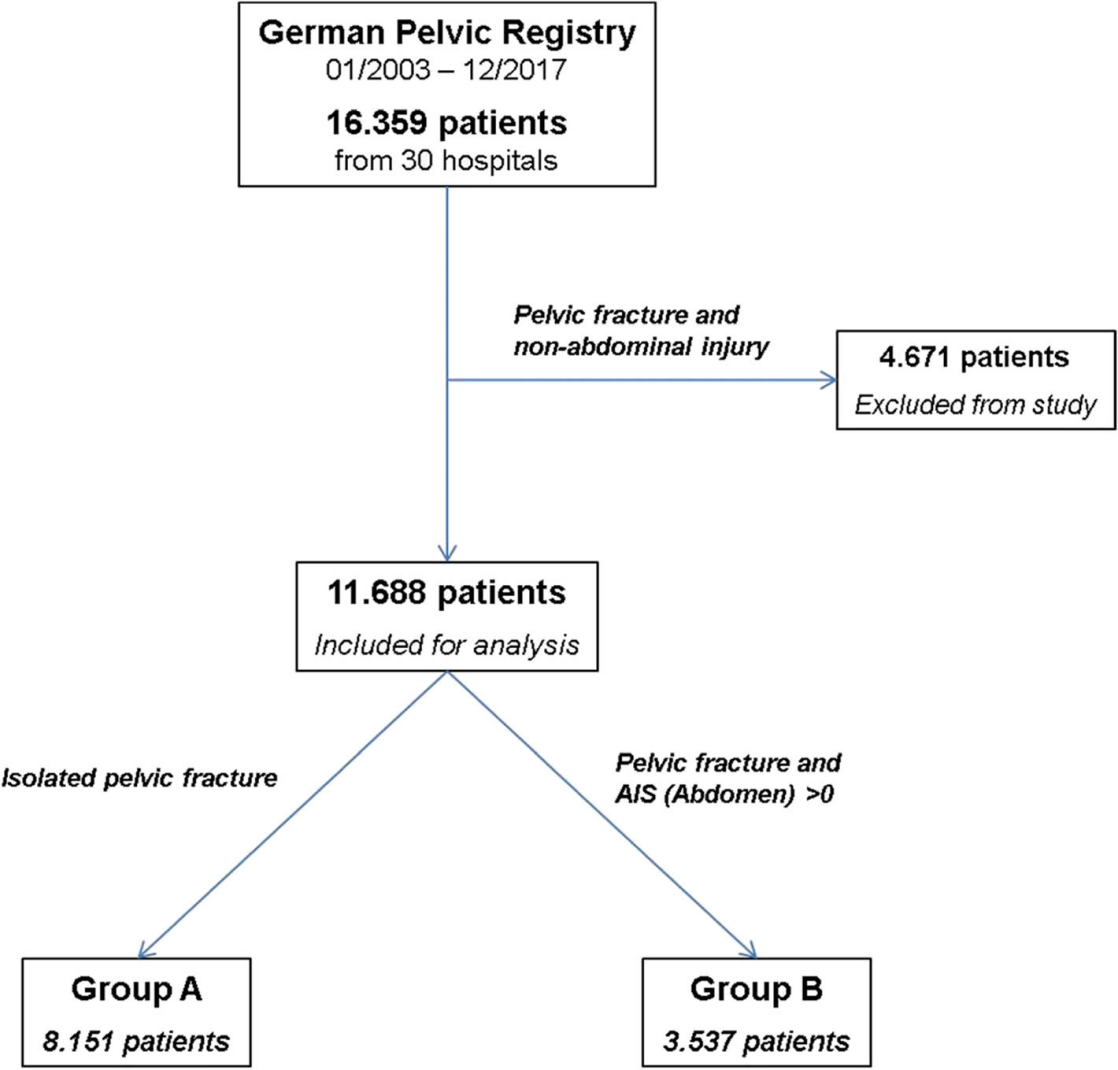
Study protocol from the German Pelvic Registry. Group A consists of patients with an isolated pelvic fracture. Group B consists of patients with a combined pelvic fracture and abdominal injury. The remaining 4.296 patients were excluded from the study. The abdominal injury was defined as an AIS (Abdomen) > 0

Of the 16.359 patients, 8.151 patients (49.8%, group A) had an isolated pelvic fracture with either a pelvic ring and/or an acetabular fracture and 3.537 patients (21.6%, group B) had a combined injury with a pelvic fracture and an abdominal injury. 4.671 patients (28.6%) were excluded due to a combined injury with a pelvic fracture and non-abdominal injuries (Fig. 1)

**Fig. 2:**
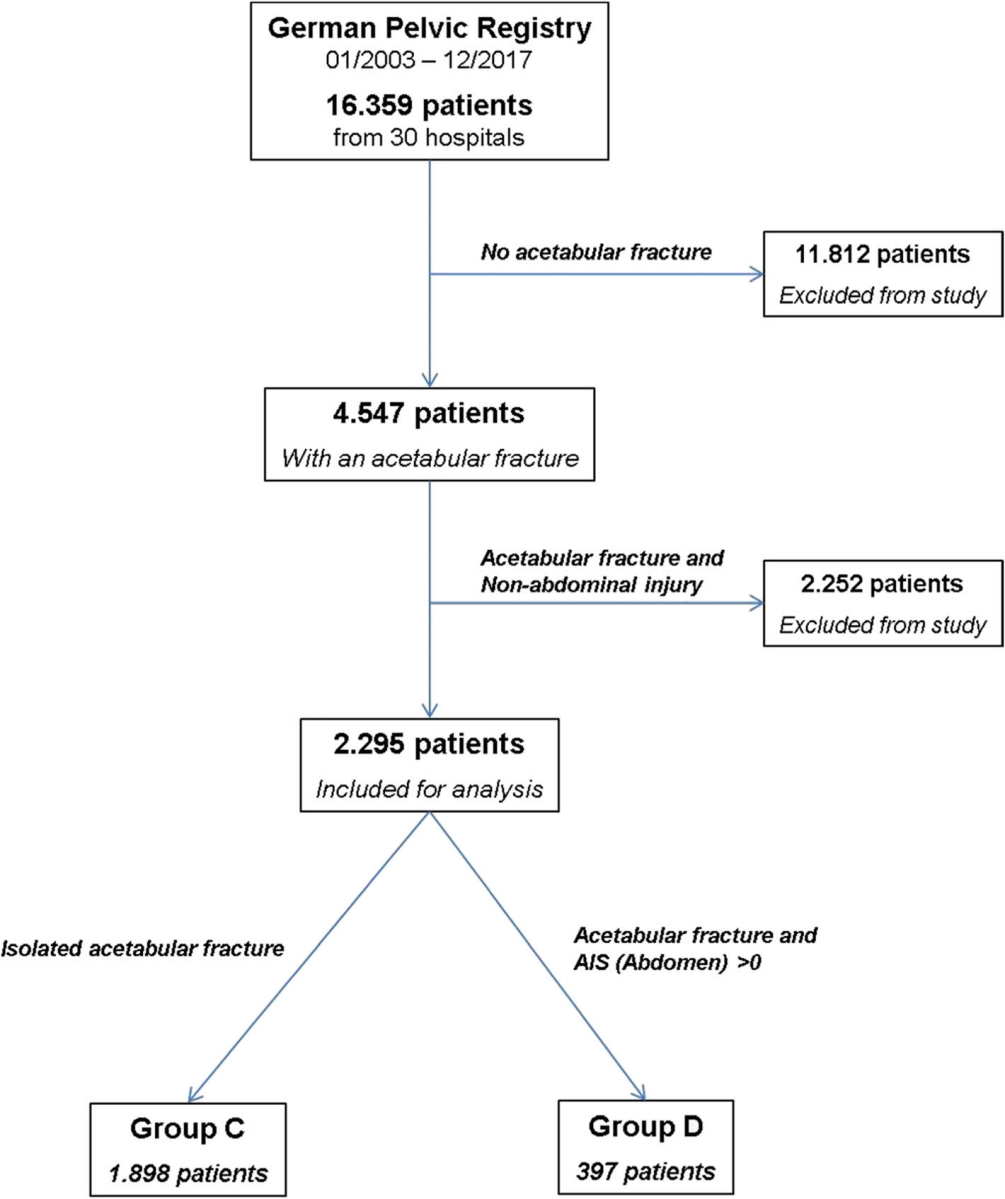
Study protocol for the acetabular fractures from the German Pelvic Registry. Group C consists of patients with an isolated acetabular fracture. Group D consists of patients with a combined acetabular fracture and an abdominal injury. The remaining 14.006 patients were excluded from the study. The abdominal injury was defined as an AIS (Abdomen) > 0

Of 4.547 acetabular fractures in the GPR, 1.898 (41.8%, group C) had an isolated acetabular fracture and 397 (8.7%, group D) had a combined injury with an acetabular fracture and an abdominal injury. 2.252 patients (49.5%) were excluded due to a combined injury with an acetabular fracture and non-abdominal injuries (Fig. 2).

### Basic data and fracture distribution

Patients with an isolated pelvic fracture (group A) were significantly older than patients with a combined pelvic and abdominal injury (group B). The gender distribution was shifted towards more male patients in group B. Moreover, the ISS in Group B was significantly higher than that in group A.

The main fracture type in both groups was pelvic ring fractures with around 75% of the fractures. The fracture distribution (isolated pelvic ring fracture, isolated acetabular fracture, or combined pelvic ring and acetabular fracture) was equal in both groups.

However, while in group A, the rate of unstable pelvic ring fractures (Tile B or C) was around 55%; the rate increased in group B to 80% (*p* < 0.001) (Table 1).

**Table 1:** Comparison of the demographic data and fracture distribution between patients with an isolated pelvic fracture (group A) and a combined abdominal/pelvic injury (group B)

	Group A	Group B	*p* value
Number (*n*)	8.151	3.537	
Age (years)	70.5 ± 20.4 [4–105]	47.3 ± 22.0 [12–92]	< 0.001 ^#^
Gender			< 0.001 *
Male (*n*)	35.5% (2.893)	62.1% (2.195)	
Female (*n*)	65.5% (5.258)	37.9% (1.342)	
ISS	9 (5)	26 (17)	< 0.001 ^§^
Type of pelvic fracture			0.28
Pelvic ring fracture	73.1% (5.956)	74.9% (2.650)	
Acetabular fracture	23.3% (1.898)	11.2% (397)	
Combined pelvic ring + acetabular fracture	3.6% (297)	13.9% (490)	
Type of pelvic ring fracture			< 0.001 *
Stable (Tile A)	44.8% (2.669)	20.0% (530)	
Unstable (Tile B/C)	55.2% (3.287)	80.0% (2.120)	

*ISS*, Injury Severity Score

The data of the ISS are given as median and IQR.

*Mann-Whitney *U* test (isolated pelvic fracture vs. combined injury)

^§^Median-test (isolated pelvic fracture vs. combined injury)

^#^Student’s *t* test (isolated pelvic fracture vs. combined injury)

### Clinical course

Regarding the hemodynamical status of the patients at admission, valid data for 49 patients in group A and for 705 patients in group B were available. Hemodynamically unstable (Hb < 8.0 g/dl and/or RR_syst_ < 100 mmHg) were around 12–17% in patients with an isolated pelvic fracture and 24–32% in patients with a combined injury (*p* < 0.05).

Patients in group B underwent surgery significantly more often for the pelvic injury (53.4% vs. 29.9%; *p* < 0.001) and had more emergency pelvic stabilizations (34.4% vs. 6.7%; *p* < 0.001) than patients in group A.

While the mean time until emergency stabilization was significantly longer in group A (113 ± 97 vs. 76 ± 76 min; *p* < 0.001), the mean time until definitive stabilization of the pelvic fractures was not different (5.4 ± 8.0 vs. 5.2 ± 5.5 days).

The mean time of treatment was nearly doubled in the group of the combined injury compared to the group of isolated pelvic fracture (27 ± 25 vs. 13 ± 14 days; *p* < 0.001).

Also, the overall complication, the rate of osteosynthesis-associated complications, and the mortality rate were significantly higher in group B compared to group A (Table 2).

**Table 2:** Comparison of the clinical course between patients with an isolated pelvic fracture (group A) and patients with a combined abdominal/pelvic injury (group B). Patients in group B were operated significantly more often due to their pelvic fracture. The time until emergency stabilization was shorter in group B, while the time until definitive pelvic surgery was longer. The clinical course was significantly prolonged with increased rates of morbidity and mortality.

	Group A	Group B	*p* value
Number (*n*)	8.151	3.537	
Hemodynamical status at admission			< 0.001*
Hb <8.0g/dl	12.2 % (6/49)	24.7 % (174/705)	
RRsyst. <100mmHg	17.1% (7/41)	32.7 % (224/686)	
Operative pelvic stabilization			
Emergency stabilization	6.7 % (547)	34.4 % (1.216)	<0.001*
Definitive pelvic fixation	29.9 % (2.440)	53.4 % (1.888)	<0.001*
Time until emergency stabilization (min)	113 ± 97 [2 – 420]	76 ± 76 [2 – 406]	<0.001^#^
Time until definitive fixation (days)	5.4 ± 8.0 [0 – 42]	5.2 ± 5.5 [0 – 43]	0.19^#^
Clinical course			
Length of hospital stay (days)	13 ± 14 [0 – 213]	27 ± 25 [0 – 287]	<0.001^#^
Overall morbidity	9.9 % (805)	21.9 % (776)	<0.001*
Osteosynthesis-associated morbidity	7.6 % (186)	10.6 % (201)	0.001*
Overall mortality	1.9 % (157)	8.0 % (287)	<0.001*

*Mann-Whitney-*U*-test (isolated pelvic fracture vs. combined injury)

^#^Student`s *t*-test (isolated pelvic fracture vs. combined injury)

### Quality of surgery in acetabular fractures

Regarding intraoperative data, the surgical time was significantly shorter in group C compared to group D (176 ± 81 vs. 198 ± 104 min, *p* = 0.001), while the intraoperative blood loss was not different.

Despite the preoperative fracture step-off was slightly larger in group D, there was no difference in the postoperative fracture step-off between the two groups. Regarding the Matta grading, there was no difference between the two groups (Table 3).

**Table 3:** Clinical and surgical outcome of patients with isolated acetabular fractures (group C) and with a combined acetabular/abdominal injury (group D). Group D patients underwent significantly more often an emergency stabilization of the acetabular fracture. Definitive surgery was not different in both groups but the patients in group D were later operated. While the patients in group D were treated significantly longer and had a higher overall complication rate, there was no difference regarding the osteosynthesis-associated complications

	Group C	Group D	*p* value
Number (*n*)	1.898	397	
Duration of surgery (min)	176 ± 81 [60–760]	198 ± 104 [60–723]	< 0.001^#^
Blood loss (ml)	600 ± 511 [100–3000]	660 ± 514 [100–3000]	0.46
Step preoperatively (mm)	7.6 ± 8.1 [0–160]	8.0 ± 13.8 [0–160]	0.01^#^
Step postoperatively (mm)	1.2 ± 2.5 [0–33]	1.1 ± 2.2 [0–25]	0.28
Quality of reduction by Matta score			0.39
Grade 1: 0–2 mm residual step (anatomical)	84.0 % (982)	85.3 % (221)	
Grade 2: 2–3 mm residual step (imperfect)	4.9 % (57)	6.6 % (17)	
Grade 3: > 3 mm residual step (poor)	8.5 % (100)	6.2 % (16)	
No postoperative data available	2.6 % (30)	1.9 % (5)	

*Mann-Whitney *U* test (isolated acetabular fracture vs. combined injury)

^#^Student’s *t* test (isolated acetabular fracture vs. combined injury)

## Discussion

The treatment of polytraumatized patients improves significantly with the implementation a standardized emergency treatment, e.g., according to the ATLS©-protocols (Advanced Trauma Life Support©). So, potentially life-threatening bleedings can be detected early and thus leads to improved survival [15]. A multi- and interdisciplinary team of trauma surgeons, general or visceral surgeons, (interventional) radiologists, anesthesiologists, and intensive care physicians should discuss the therapy regime together. In case of a polytraumatized patient, the trauma team has to decide which treatment is most urgent and whether initiation of damage control surgery or damage control orthopedic surgery is indicated [16, 17]. In consequence, if a combined abdominal injury and pelvic fracture occurs, the abdominal injury is most common decisive, and fractures should be temporarily stabilized by an external fixator if stabilization is necessary. However, despite the best time-period for definitive fracture stabilization is usually within the first few days, in polytraumatized patients, it often has to wait until approval of the visceral surgeons regarding the abdomen and the intensive care physicians regarding the general condition to prevent a so-called “second-hit” to the patient.

Besides factors like instability or grade of dislocation, the treatment strategy of pelvic ring fractures or acetabular fractures also depends on concomitant injuries. While many pelvic fractures can be treated conservatively, pelvic ring fractures with involvement of the posterior pelvic ring and dislocated acetabular fractures usually require surgical reduction and osteosynthetic fixation. As mentioned, the optimal time for surgical treatment is being discussed. If the patient’s status allows for it and an adequate surgical experience for pelvic fractures is available, a definitive treatment within the first 24 h after the accident is possible with good clinical and surgical results [18]. However, especially in cases of high-energy traumata, there are often concomitant injuries evident like associated abdominal injuries which may result in a delayed osteosynthetic fixation of pelvic fractures. A delay over three weeks has been proven to go along with a worse surgical outcome regarding reduction quality [19].

The mortality of pelvic fractures in the literature is about 6–13% with decreasing rates in the last decades [20–23]. The main cause of death in pelvic fractures is major bleeding either from fractures or from concomitant injuries. By implementing standardized prehospital and emergency department trauma management strategies (e.g., ATLS®), including non-invasive stabilization of the pelvis (e.g., pelvic binder), as well as aggressive transfusion regimes with early use of blood products and coagulation factors, the rates of severe bleeding and exsanguination in pelvic fractures could be reduced significantly [24–28]. However, the therapeutic treatment of pelvic fractures still depends on both the hemodynamic status and concomitant diseases. There is consensus for unstable pelvic ring fractures with hemodynamic instability. These fractures usually undergo emergency stabilization using an external fixator (for the anterior pelvic ring) or the pelvic C-clamp (for the posterior pelvic ring). If the bleeding cannot be controlled by these procedures alone, either an interventional radiological embolization (in hemodynamically stable patients) or a surgical approach to control the bleeding by preperitoneal pelvic packing (in hemodynamically unstable patients) is possible [29, 30].

The optimal time of definitive surgical stabilization of a pelvic ring or acetabular fracture is difficult to find. The impact of multidisciplinary approach leads to improvement in performance and in patient outcomes. The main parts of these issues are a massive hemorrhage protocol, a decision-making algorithm, and employment of specialist pelvic orthopedic surgeons with significant improvements in the targeted processes of care [31]. If immediate arteriography and angioembolisation of bleeding pelvic vessels is unavailable, delayed or the existence of additional major injuries require treatment (i.e., head, chest, intra-abdominal, long bone) and external fixation and pelvic packing can be used to further reduce pelvic venous bleeding [32]. As mentioned above, definitive treatment within 24 h after the accident is associated with good clinical and radiological results. However, this is often not possible due to either concomitant injuries or the missing surgical experience for pelvic orthopedic surgery. The decision-making process in the timing of surgical interventions has to be taken into consideration, that in the vulnerable trauma patients the delay of bone fracture fixation leads to increasing morbidity and prolonged immobilization [33]. Decision-making in acute trauma care has also—besides the above mentioned medical reasons—to consider the capacity limits of intensive care units and should also intend to strengthen the trauma care line. Therefore, an efficient trauma management leads to reduced consumption of clinical resources and cost reduction with even better patient outcome with fewer complications and shorter length of hospital stay [34]. The study showed that after fast resuscitation within 36 h the treatment delays were in most of the cases because of nonmedical reasons. Therefore, definitive stabilization of a pelvic fracture is often delayed. There are studies comparing different points of time. While the early definitive treatment (2–4 days after accident) results in an increased morbidity rate, the morbidity rate decreases significantly, if surgery is performed 5–8 days after the accident [18]. However, another recent study showed that definitive fracture fixation, including fixation of long bones, pelvic ring or acetabular fractures, and spinal fusions, even in the presence of an open abdomen can be performed safely and is associated with a significant decrease in clinically relevant surgical site infections, compared with delayed fracture fixation until abdominal wall closure. Therefore the time delay while awaiting abdominal wall closure is unjustified [35].

Another decisive factor in diagnosing and treating patients with a pelvic fracture is the presence of associated abdominal injuries. The rate of associated abdominal injuries in pelvic fractures is about 15% [36]. In our cohort, 21.6% of the patients had a concomitant abdominal injury. Another U.S. study showed that 16.5% of the patients with pelvic fractures had concomitant abdominal or urogenital injuries. Solid organs were involved in 11.8% of the cases (liver 6.1%, spleen 5.2%); gastrointestinal perforations affected more often the small bowel than the large bowel. Traumatic aortic injuries were rare (1.4%). In minor pelvic fractures, urogenital injuries outweighed liver injuries [37]. The clinical course in patients with a combined abdominal/pelvic injury was significantly prolonged with increased rates of morbidity and mortality. Most likely, this can be attributed to the more serious injury pattern—indicated by a higher ISS score [38].

However, in our cohort, neither the postoperative results regarding the osteosynthetic fixation of an acetabular fracture nor the osteosynthesis-associated complications are affected by a concomitant abdominal injury. Indeed, the time until definitive surgical treatment is delayed in patients with a combined abdominal/pelvic injury. However, with a mean time frame of almost 6 days after the accident, the definitive surgical treatment still occurs within the recommended 5–8 days after accident [28].

The high number of patients and the multicenter registry are the main strengths of this study, resulting in a good validity regarding clinical and operative outcomes of the pelvic and acetabular fractures. Of course, there is a natural bias in the nature of registries, that the accuracy of some parameters (e.g. more often postoperative CT scans to measure the remaining postoperative fracture step) might change over the time and need future adjustments. One major weakness beside the retrospective character is the fact that the main focus of the German Pelvic Registry is the treatment of pelvic fractures. Therefore there are limitations of this study regarding the associated abdominal injury. For a firm assessment of different abdominal injuries on the quality of care of pelvic fractures, a specific prospective registry study would be necessary.

## Conclusion

In conclusion, despite a delay in the definitive surgical treatment of pelvic fractures due to associated abdominal injuries, the clinical outcome of the pelvic fractures and especially the quality of reduction in acetabular fractures are not affected in a negative way. Increased rates of overall morbidity and mortality as well as prolonged inpatient treatment can be attributed to the more severe injuries. Especially in patients with combined abdominal/pelvic injuries, the optimal time of the definitive surgical treatment of the pelvic fractures must be found in an interdisciplinary discussion to achieve the best possible fracture reduction quality together with low morbidity rates.

## Data Availability

Not applicable

## Abbreviations

AIS: Abbreviated Injury Score
AO: Arbeitsgemeinschaft für Osteosynthesefragen
ATLS: Advanced Trauma Life Support
CT: Computer-Tomography
DGU: Deutsche Gesellschaft für Unfallchirurgie (German Society for Traumatology)
GPR: German Pelvic Registry
ISS: Injury Severity Score
U.S.: United States
